# Effects of NS2B-NS3 protease and furin inhibition on West Nile and Dengue virus replication

**DOI:** 10.1080/14756366.2017.1306521

**Published:** 2017-04-07

**Authors:** Jenny Kouretova, M. Zouhir Hammamy, Anton Epp, Kornelia Hardes, Stephanie Kallis, Linlin Zhang, Rolf Hilgenfeld, Ralf Bartenschlager, Torsten Steinmetzer

**Affiliations:** a Department of Pharmacy, Institute of Pharmaceutical Chemistry, Philipps University, Marburg, Germany;; b German Center for Infection Research (DZIF), University of Marburg, Marburg, Germany;; c Department of Infectious Diseases, Molecular Virology, Heidelberg University, Heidelberg, Germany;; d Institute of Biochemistry, Center for Structural and Cell Biology in Medicine, University of Lübeck, Lübeck, Germany;; e German Center for Infection Research (DZIF), University of Lübeck, Lübeck, Germany;; f German Center for Infection Research (DZIF), Heidelberg University, Heidelberg, Germany

**Keywords:** West Nile virus, Dengue virus, NS2B-NS3 protease, furin, antiviral activity

## Abstract

West Nile virus (WNV) and Dengue virus (DENV) replication depends on the viral NS2B-NS3 protease and the host enzyme furin, which emerged as potential drug targets. Modification of our previously described WNV protease inhibitors by basic phenylalanine analogs provided compounds with reduced potency against the WNV and DENV protease. In a second series, their decarboxylated P1-*trans*-(4-guanidino)cyclohexylamide was replaced by an arginyl-amide moiety. Compound 4-(guanidinomethyl)-phenylacetyl-Lys-Lys-Arg-NH_2_ inhibits the NS2B-NS3 protease of WNV with an inhibition constant of 0.11 µM. Due to the similarity in substrate specificity, we have also tested the potency of our previously described multibasic furin inhibitors. Their further modification provided chimeric inhibitors with additional potency against the WNV and DENV proteases. A strong inhibition of WNV and DENV replication in cell culture was observed for the specific furin inhibitors, which reduced virus titers up to 10,000-fold. These studies reveal that potent inhibitors of furin can block the replication of DENV and WNV.

## Introduction

West Nile virus (WNV) and Dengue virus (DENV) are mosquito-transmitted pathogenic flaviviruses. DENV is prevalent in most tropical and subtropical areas of the world. It was estimated that the four serotypes of DENV infect approximately 390 million people each year^1^. In severe cases, infections are associated with dengue hemorrhagic fever and dengue shock syndrome. In contrast, WNV infections mostly cause only mild flu-like symptoms. However, a higher rate of severe neurologic diseases occurred during outbreaks in Eastern Europe since the 1990s, and WNV has finally moved into the focus of the public in 1999, after the virus has spread to New York City, across the USA, and to neighboring countries^2^. Meanwhile, a veterinary vaccine against WNV infections of horses became available, but no WNV vaccine for use in humans has been approved, so far^3^. In December 2015, a first tetravalent DENV vaccine developed by Sanofi Pasteur (Dengvaxia^®^) was introduced in Mexico, the Philippines, and Brazil, although only partial protection with variations between the different serotypes could be achieved^4^. Additional DENV vaccines are presently in development^5^^,^^6^. So far, there is no specific antiviral treatment against WNV and DENV infections available^7^^,^^8^ and the presently most effective protection is simply to avoid the bites of virus-transmitting mosquitoes.

Flaviviruses contain a single-stranded positive-sense RNA genome, which serves as mRNA for the translation of a single immature polyprotein precursor^2^. Multiple cleavages of the precursor provide the structural and nonstructural proteins required for new virus progeny. The proteolytic activity of WNV and DENV is located in the N-terminal part of its NS3 protein, which is associated to the cytosolic site of the endoplasmatic reticulum via noncovalent interactions to a central hydrophilic domain of the integral membrane protein NS2B^9^. Due to the dependency of flavivirus replication on polyprotein processing, the viral protease emerged as a potential target for the treatment of flavivirus infections^10^^,^^11^. It is a typical serine protease containing a catalytic triad consisting of residues Ser135, His51, and Asp75. Various X-ray structures of covalently linked artificial NS2B-NS3 constructs of the WNV and DENV proteases are available, which were recently reviewed^9–11^. The homology of the WNV NS2B 40-amino acid cofactor together with the NS3 protease domain compared to the analogous DENV-2 domains is 66.5%^12^, both enzymes preferably cleave substrates after two basic residues in the P2–P1 positions^13^.

A similar preference for Arg in the P1 position and a second basic P2 residue is known for the host protease furin, which is also essential for flavivirus propagation. It belongs to the family of proprotein convertases (PCs) and contains a subtilisin-like Ca^2+^-dependent serine protease domain^14^. In the *trans*-Golgi compartment furin catalyzes the essential removal of the pr segment from the prM precursor of the membrane glycoprotein M, which interacts with the viral envelope glycoprotein E. The cleaved pr peptide remains associated as long as progeny virions are exposed to neutral pH in the extracellular space, where it dissociates providing fusion-competent infectious virus particles^15^. Despite a more extended specificity in the non-primed part of substrates for furin, which strongly prefers additional arginine residues in P4 and/or P6 position, there might be some cross-potency between substrate-analog active-site inhibitors of flavivirus NS2B-NS3 proteases and of furin, which indeed, was previously found for a series of poly-d-Arg peptides^16^.

**Figure 1.**
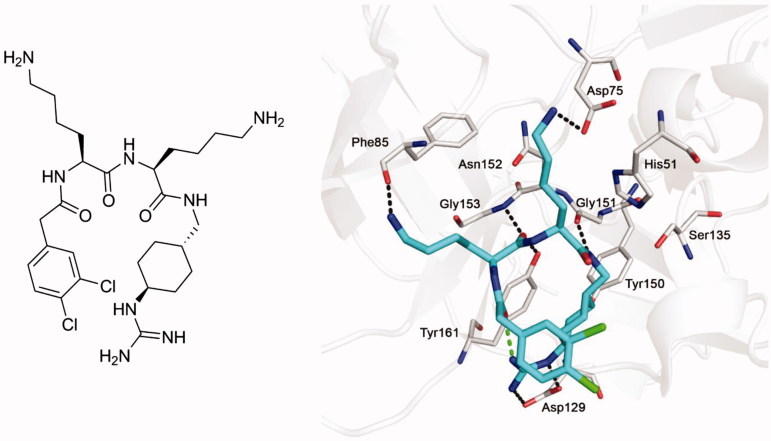
Structure of inhibitor **1** (left panel) and its bound conformation in the active site of the WNV NS2B-NS3 protease (right panel, PDB: 2YOL^19^). Intermolecular polar contacts between enzyme and inhibitor are shown as dashed lines in black, the intramolecular contact stabilizing the horseshoe-like inhibitor conformation is shown in green.

In previous work, various substrate-analog inhibitors of the NS2B-NS3 protease of WNV containing decarboxylated arginine mimetics as P1 residues have been identified^17–19^. One of the most potent derivatives from our series was inhibitor **1** (*K*_i_= 0.13 µM), which contains a *trans*-(4-guanidino)cyclohexylamide (GCMA) as P1 residue. The inhibitor adopts a horseshoe-like conformation when bound to the active site of the WNV protease (PDB: 2YOL), resulting in a close proximity between the inhibitor’s N-terminal phenylacetyl group and the guanidinocyclohexyl residue in P1 position (Figure 1)^19^.

The crystal structure revealed a relatively large and open S3 binding site; only a single hydrogen bond between the terminal amino group of the P3 lysine residue and the carbonyl of Phe85 was found. This suggested to replace the flexible lysine in that position by more bulky basic substituted phenylalanine analogs and to use these unnatural amino acids also in the P2 position. Similar phenylalanines were recently incorporated into a series of peptidic inhibitors containing a C-terminal lysine-phenylglycine amide. Docking studies suggested that this dipeptide segment occupies the S1–S1′ region, whereas the 4-amidino- or 4-guanidinophenylalanines are placed in the S2 pocket^20^. In a second approach, we have incorporated the P4–P2 segment of inhibitor **1** in peptidic inhibitors with arginine in the P1 position. Interestingly, some of these simple linear peptides showed a relatively potent inhibition of the WNV protease. Moreover, due to the similarity in substrate specificity, we have also tested the potency of previously described and two new multibasic furin inhibitors against the WNV and DENV NS2B-NS3 proteases. Selected inhibitors were used in a cell culture assay for the inhibition of DENV and WNV propagation. The results of this work are described in this paper.

## Materials and methods

Reagents for synthesis, including protected standard amino acids, coupling reagents and solvents were obtained from Bachem (Bubendorf, Switzerland), Iris Biotech GmbH (Marktredwitz, Germany), Alfa Aesar (Karlsruhe, Germany), Acros Organics-Fisher Scientific (Schwerte, Germany), Merck-Millipore (Darmstadt, Germany), and Sigma-Aldrich (Taufkirchen, Germany). The building blocks *trans*-1-(Cbz-amino)-4-aminomethyl-cyclohexane × HCl and Fmoc-4-aminomethyl-phenylacetic acid were purchased from Iris Biotech GmbH (Marktredwitz, Germany) and Polypeptides (Strasbourg, France), respectively.

Analytical HPLC experiments were performed on a Shimadzu LC-10A system (column: Nucleodur C_18_, 5 µM, 100 Å, 4.6 mm× 250 mm, Macherey-Nagel, Düren, Germany) with a linear gradient of acetonitrile (solvent B) and water (solvent A) both containing 0.1% TFA at a flow rate of 1 ml/min (1% increase of solvent B per min), detection at 220 nm. The final inhibitors were purified by preparative HPLC (pumps: Varian PrepStar Model 218 gradient system, detector: ProStar Model 320 with detection at 220 nm, fraction collector: Varian Model 701; column: C_8_, Nucleodur, 5 µM, 100 Å, 32 × 250 mm, Macherey-Nagel) by a linear gradient (0.5% increase of solvent B per min) with the same solvents as described above at a flow rate of 20 ml/min. All final inhibitors were obtained as TFA-salts after lyophilization. The molecular mass of the synthesized compounds was determined using a QTrap 2000 ESI spectrometer (Applied Biosystems, now Life Technologies, Carlsbad, CA).

### Synthesis

**Figure 2.**
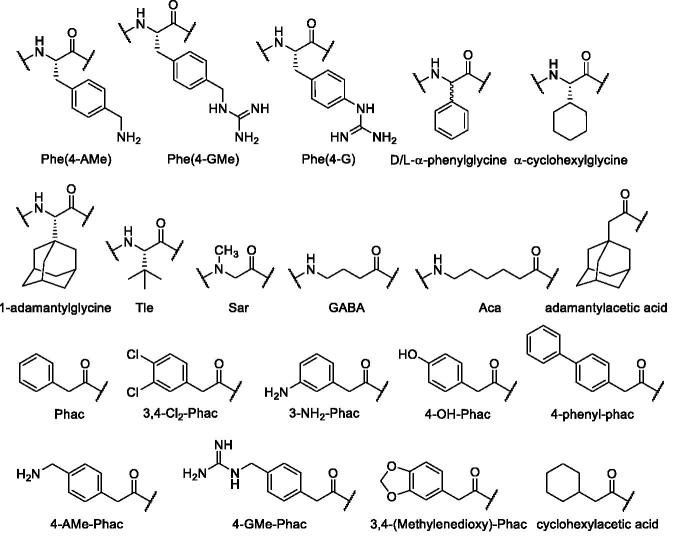
Structures and used abbreviations of unusual amino acids and acyl residues.

All compounds were prepared by a combination of solid phase and solution synthesis or by pure solid phase peptide synthesis (SPPS). The structures and used abbreviations of unusual amino acids and acyl residues are shown in Figure 2.

**Scheme 1.**
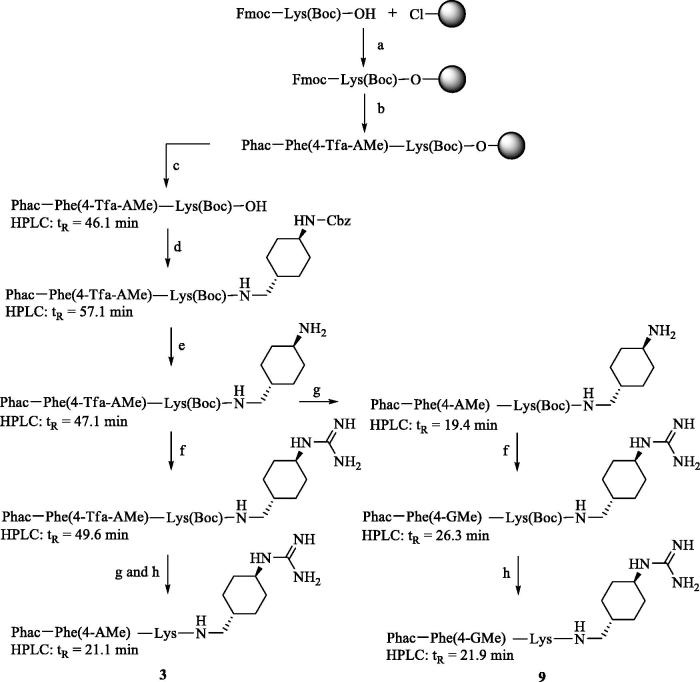
Synthesis of inhibitors **3** and **9**. HPLC analysis, used for monitoring the reactions, started at 10% solvent B. (a) Loading of 2-chlorotrityl chloride resin, Fmoc-Lys(Boc)-OH, 4 equiv. DIPEA in dry DCM, 2 h; (b) Manual Fmoc SPPS with 3 equiv. Fmoc-Phe(4-Tfa-AMe)-OH or phenylacetic acid, 3 equiv. HBTU and 6 equiv. DIPEA; Fmoc removal with 20% piperidine in DMF; (c) 1% TFA in DCM, 3 × 30 min; (d) 1 equiv. *trans*-1-(Cbz-amino)-4-aminomethyl-cyclohexane × HCl, 1 equiv. PyBOP, 3 equiv. DIPEA, DMF; (e) H_2_ and Pd/C as a catalyst in 90% acetic acid, stirring overnight at r.t.; (f) 3–6 equiv. 1*H*-pyrazole-1-carboxamidine × HCl, 4 equiv. DIPEA in DMF, 16 h; (g) 1 M NaOH in dioxane/water, pH 12 at r.t. 3 h, neutralization by 10% TFA; (h) 90% TFA, at r.t. 1 h, preparative HPLC. All HPLC measurements of intermediates started at 10% solvent B, the analysis of the more hydrophilic final inhibitors **3** and **9** started at 1% solvent B.

The analogs of the first series (Table 1) contain a constant P1 GCMA group and the lysine residues in P3 and P2 position of the reference inhibitor **2**^19^ were replaced by basic substituted phenylalanine derivatives. The side-chain-protected P4-P2 segment was synthesized on a 2-chlorotrityl chloride resin (initial loading 1.5 mmol/g, Iris Biotech GmbH, Marktredwitz, Germany). The unnatural aminomethylene-substituted phenylalanine derivatives Fmoc-Phe(4-Tfa-AMe)-OH and Fmoc-Phe(3-Tfa-AMe)-OH, protected by a trifluoroacetyl (Tfa) group, were obtained from their previously described Nα-Boc-protected analogs^21^. Their Boc group was removed by treatment with TFA and the obtained intermediates were reacted with Fmoc-OSu. As an example, Scheme 1 shows the strategy for the synthesis of inhibitors **3** and **9**, containing 4-aminomethylenephenylalanine (Phe(4-AMe)) and the analogous 4-guanidinomethylenephenylalanine (Phe(4-GMe)) as P3 residue, respectively.

**Table 1.** Analytical data and inhibition of the WNV and DENV NS2B-NS3 proteases by inhibitors of the formula.


No.	P3^a^	P2^a^	MS (calc./found) (M + H)^+^	HPLC^b^*t_*R*_* (min)	*K*_i_*(*μM) (WNV)	% Inhib.^c^ (DENV)
**2**^d^	Lys	Lys	544.4/545.3	20.5	1.2	n.d.^a^
**3**	Phe(4-AMe)^a^	Lys	592.4/593.2	21.1	4.71	12.4
**4**	Lys	Phe(4-AMe)	592.4/593.1	23.1	28.9	n.d.
**5**	Phe(4-AMe)	Phe(4-AMe)	640.4/641.2	24.1	13.6	10.8
**6**	Phe(3-AMe)	Lys	592.4/297.4^e^	23.3	22.2	n.d.
**7**	Lys	Phe(3-AMe)	592.4/593.3	23.9	134	n.d.
**8**	Phe(3-AMe)	Phe(3-AMe)	640.4/641.4	25.8	44.2	5.5
**9**	Phe(4-GMe)	Lys	634.4/635.1	21.9	44.0	n.d.
**10**	Lys	Phe(4-GMe)	634.4/635.2	22.4	75.6	n.d.
**11**	Phe(4-GMe)	Phe(4-GMe)	724.4/725.3	26.9	85.3	17.1
**12**	Phe(3-GMe)	Lys	634.4/635.2	26.4	29.6	31.1
**13**	Lys	Phe(3-GMe)	634.4/635.3	24.5	99.4	n.d.
**14**	Phe(3-GMe)	Phe(3-GMe)	724.4/725.3	28.5	21.6	n.d.
**15**	Lys	Phe(4-G)	620.4/311.3^e^	25.2	18.2	14.7
**16**	Phe(4-G)	Lys	620.4/311.5^e^	24.9	29.8	n.d.

a See Figure 2 for abbreviation; n.d.: not determined.

b HPLC measurements started at 1% solvent B.

c % Inhibition at 100 μM inhibitor in the presence of 125 μM substrate concentration.

d Reference compound available from previous studies^19^.

e MS found (M + 2H)^2+^/2.

The crude side-chain-protected intermediate Phac-Phe(4-Tfa-AMe)-Lys(Boc)-OH (HPLC: *t*_R_ = 46.1 min, start at 10% solvent B) was prepared by manual SPPS on 2-chlorotrityl chloride resin (100 mg) in a 2-ml polypropylene syringe with polyethylene frit (MultiSynTech, Witten, Germany) using a standard Fmoc-protocol, followed by mild acidic cleavage using 1% TFA in DCM. The following reactions (Scheme 1) were monitored by analytical HPLC, the obtained crude intermediates were not purified. All final inhibitors were purified by preparative HPLC to more than 95% purity (based on detection at 220 nm).

For the synthesis of the 4-guanidinophenylalanine-containing inhibitors **15** and **16,** Fmoc-Phe(4-NO_2_)-OH was used during SPPS, which was performed as described in Scheme 1. Mild acidic cleavage from resin provided the side-chain-protected P4-P2 intermediate, which was coupled with *trans*-1-(Cbz-amino)-4-aminomethyl-cyclohexane × HCl. Hydrogenation according to step e in Scheme 1 provided the 4-aminophenylalanine intermediates. In these cases, the conversion into the guanidine analogs was performed with the more reactive *N*,*N*′-di-Boc-1*H*-pyrazole-1-carboxamidine^22^.

The peptides **17**–**44** of the second series were synthesized on Rink-amide resin (Iris Biotech GmbH, Marktredwitz, Germany) by automated solid-phase peptide synthesis on a Syro 2000 instrument (MultiSynTech GmbH, Witten, Germany). The synthesis was performed by a standard Fmoc-protocol with double couplings using a 4-fold excess of Fmoc amino acid, HOBt and HBTU, respectively, and 8 equiv. of DIPEA. A solution of 20% piperidine in DMF was used for removal of the Fmoc group. The synthesis of the α-phenylglycine-containing inhibitors **39** and **40** resulted in a complete racemization of this residue as shown by a double peak in HPLC analysis of the final products. The racemization of phenylglycine containing peptides prepared by standard Fmoc-SPPS has been previously described^23^.

The previously described furin inhibitors **45** and **46**^24^^,^^25^ were resynthesized by a slightly modified procedure, which was also used for the preparation of the new analogs **47** and **48**. Their P5–P2 segment containing an N-terminal Fmoc-4-aminomethyl-phenylacetyl residue was synthesized on 2-chlorotrityl chloride resin. After Fmoc-removal, the terminal amino group was converted into a guanidine by treatment with *N*,*N*′-di-Boc-1*H*-pyrazole-1-carboxamidine and DIPEA^22^. After mild acidic cleavage (1% TFA in DCM) from resin, the unprotected 4-amidinobenzylamine ×2 HCl was coupled in solution, followed by strongly acidic side chain deprotection in the final step.

### Enzyme kinetic measurements

All measurements were performed at room temperature in black flat-bottom 96-well plates (Nunc, Langenselbold, Germany) using microplate readers (Tecan Safire^2^, *λ*_ex_ = 380 nm and *λ*_em_ = 460 nm, Tecan Group Ltd., Männedorf, Switzerland or Fluoroskan Ascent type 374, *λ*_ex_ = 355 nm and *λ*_em_ = 460 nm; Thermo Fisher Scientific, Vantaa, Finland). The assays with the flavivirus protease constructs were performed with 125 μl buffer (100 mM Tris pH 8.5 containing 20% glycerol, 0.01% Triton X-100, and the inhibitor), 50 μl substrate solution, and were started by addition of 25 μl enzyme solution (total assay volume 200 μl).

For measurements with the covalently linked WNV NS2B-NS3 protease construct (∼4 nM in assay) the substrate Phac-Leu-Lys-Lys-Arg-AMC ×3 TFA (50 µM, 100 µM and 200 µM in assay, K_M_ = 47.5 µM) was used, as described previously^19^.

The DENV serotype 2 NS2B-NS3 protease construct was prepared from an identical clone as described by D’Arcy et al.^26^ The purification of the protease was carried out in a buffer with high ion-strength and low pH value, in the absence of glycerol (20 mM Bis-Tris, 500 mM NaCl, pH 6.3), in order to prevent autolysis of the enzyme. Purification was performed by using a 5 ml HisTrapTM FF column (GE Healthcare) and subsequently, a HiLoad 16/60 SuperdexTM 75 column (GE Healthcare). The assays with the DENV-2 NS2B-NS3^pr^° (∼39 nM in well) were performed with the substrate Phac-Lys-Arg-Arg-AMC ×3 TFA (125 µM, 62.5 µM and 31.25 µM in assay, *K*_M_ = 60 µM). All *K*_i_ values were obtained from Dixon plots^27^ and are the average of at least two measurements. Due to the weak potency of most compounds against the DENV-2 NS2B-NS3 protease construct, only the percentage of inhibition at a constant inhibitor concentration of 100 µM in the presence of 125 µM substrate was determined. A stronger inhibition was observed for the chimeric furin and flavivirus protease inhibitors **47** and **48** (Table 3); only for these compounds *K*_i_ values have been determined.

**Table 3.** Chimeric furin, WNV and DENV-2 NS2B-NS3 protease inhibitors of the formula.


No.	P3	MS (calc./found) (M + H)^+^	HPLC *t*_R_ (min)	*K*_i_ (μM) (WNV)	*K*_i_ (μM) (DENV)	*K*_i_ (pM) (furin)
**45**	Val	749.46/375.91^a^	19.7	5.70	77.3	7.60
**46**	Tle	763.47/764.39	20.8	6.45	83.1	5.50
**47**	Arg	806.49/404.31^a^	16.2	0.65	11.6	56.9
**48**	Lys	778.48/390.2^a^	16.2	0.82	1.22	44.8

a MS found (M + 2 H)^2+^/2.

Measurements with recombinant human furin (0.95 nM in assay) were performed in 100 mM HEPES buffer (containing 0.2% Triton X-100, 2 mM CaCl_2_, 0.02% NaN_3_, and 1 mg/ml BSA, pH 7.0) with the fluorogenic substrate Phac-Arg-Val-Arg-Arg-AMC (5 µM, 20 µM, and 50 µM in assay) as described previously^25^.

### Plaque assay and cell viability

Inhibition of DENV propagation was determined by plaque assay as described recently^20^^,^^28^ with slight modifications. Huh-7 cells were seeded into 96-well plates and incubated overnight. The next day, cells were infected with DENV (strain 16681) at a multiplicity of infection (MOI) of 1 PFU/cell. Given concentrations of the compounds, dissolved in culture medium, were added to the cells together with the virus. After 2 h incubation at 37 °C, inocula were removed and cells were covered with fresh medium containing given concentrations of the inhibitors. Production of virus progeny was determined 48 h postinfection by titration of cell supernatants using a plaque assay on VeroE6 cells. Ribavirin was included as a reference compound.

An analogous plaque assay was employed for WNV-infected cells. Huh-7 cells were seeded into 96-well plates and incubated overnight. Cells were infected for 1 h with the WNV strain New York 99 at a MOI of 0.2 PFU/cell. Different concentrations of the compounds, dissolved in culture medium, were added to the cells together with the virus. After 1 h incubation at 37 °C, inocula were removed and cells were covered with fresh medium containing the inhibitors. Production of virus progeny was determined 48 h postinfection by titration of culture supernatants using a plaque assay on VeroE6 cells, Ribavirin was included as a reference compound. The influence of inhibitors on cell viability was determined by using CellTiter-Glo^®^ and CytoTox 96^®^ assays (Promega GmbH, Mannheim, Germany) according to the instructions of the manufacturer.

## Results

### Modifications in P2 and P3 position

In the first series, the lysine residues in the P2 and P3 positions of the reference inhibitor **2** (*K*_i_ = 1.2 µM against the WNV NS2B-NS3 construct) were replaced by basic phenylalanine analogs (Table 1). All inhibitors possess reduced potency against the WNV protease, regardless of the Phe residues being substituted with an aminomethylene or guanidinomethylene group in para- or meta-position of the phenyl ring or directly by a *para*-guanidino group. Among them, the best *K*_i_ value was found for Phac-Phe(4-AMe)-Lys-GCMA **3** with a 4-fold reduced potency of 4.7 µM. The data suggest that the replacement of the P2 Lys residue results in an even stronger drop in potency compared to modifications in the P3 position. Surprisingly, the double-substituted inhibitors **5**, **8**, **11**, and **14** gained some affinity compared to the single replacements in the P2 position (inhibitors **4**, **7**, **10**, and **13**), although a further drop in potency was expected. Moreover, a poor affinity was found for all tested inhibitors against the DENV-2 NS2B-NS3 protease.

In a related series (data not shown) containing a C-terminal agmatine and N-terminal 3,4-dichlorophenylacetyl group, additional residues such as arginine, the shorter norarginine, Homophe, and Ser(Benzyl) were used as P2 or P3 residues. However, these analogs were less potent than their reference compound 3,4-di-Cl-Phac-Lys-Lys-agmatine (*K*_i_ = 0.4 µM), which was available from our former study^19^. This confirms the previously described result^18^^,^^29^ that lysine seems to be the most suited P2 and P3 residue in substrate analog inhibitors of the WNV protease so far.

### Peptidic inhibitors with P1 arginine-amides

**Figure 3.**
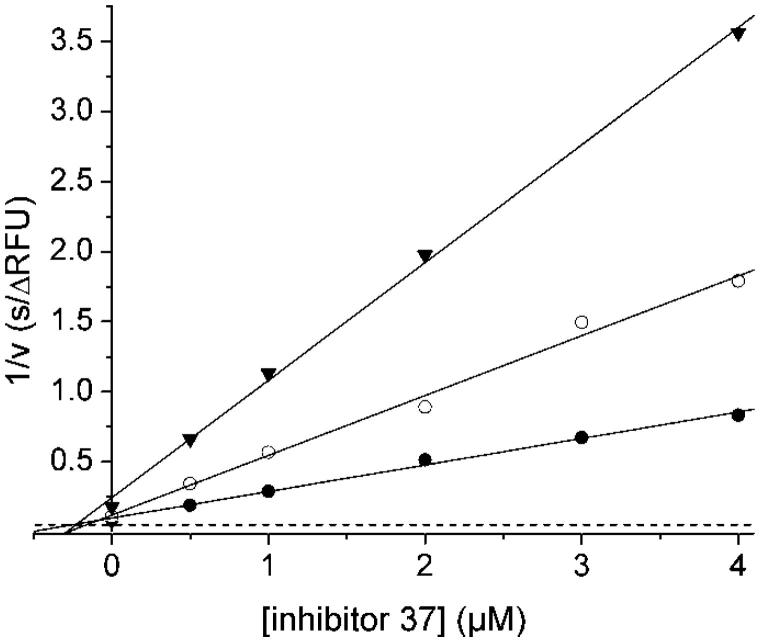
Dixon plot for the inhibition of the WNV NS2B-NS3 protease by inhibitor **37**. Kinetic measurements have been performed with three different concentrations of the substrate Phac-Leu-Lys-Lys-Arg-AMC at 100 (•), 50 (^), and 25 μM (▾) using various inhibitor concentrations. The dashed line represents 1/*V*_max_, which was obtained from a Michaelis–Menten plot determined in parallel on the same 96-well plate.

The preferred P3-P1 Lys-Lys-Arg segment^29^ was used for the preparation of a series of peptidic inhibitors. The peptides were modified in their prime site segment and at the P4 position (Table 2). A strong inhibitory potency was found for simple phenylacetyl-capped tripeptides containing a C-terminal P1 arginyl amide moiety (**27**) and for their elongated derivatives with one or two glycine residues in the P1′ and P2′ positions (**17** and **18**). Incubation experiments and subsequent HPLC analysis revealed that these compounds are relatively stable against cleavage by the WNV-protease, only a minor amount of the cleavage product Phac-Lys-Lys-Arg-OH was detected after 4 h. In contrast, under identical conditions approximately 33% of the chromogenic substrate Phac-Lys-Lys-Arg-pNA was cleaved (data not shown). Thus, these compounds seem to be poor substrates of the WNV NS2B-NS3 protease and therefore, act as competitive inhibitors. The best *K*_i_ values <0.2 µM were obtained for peptides with the general structure phenylacetyl-Lys-Lys-Arg-NH_2_ containing an N-terminal guanidinomethyl substitution at the P4-residue. Figure 3 shows the Dixon plot for the competitive reversible inhibition of the WNV protease by inhibitor **37**.

**Table 2.** Analytical data and inhibition of the WNV and DENV NS2B-NS3 protease by peptides of the formula P4-Lys-Lys-Arg-R′ (HPLC measurements started at 1% solvent B).

No.	P4^a^	R′^a^	MS (calc./found) (M + H)^+^	HPLC *t*_R_ (min)	*K*_i_ (μM) (WNV)	% Inhib. (DENV)^b^
**17**	Phac	Gly-Gly-NH_2_	661.4/662.26	16.4	1.56	35.1
**18**	Phac	Gly-NH_2_	604.4/605.4	16.9	2.56	30.7
**19**	Phac	Ala-NH_2_	618.39/619.27	17.0	25	5.6
**20**	Phac	DAla-NH_2_	618.39/619.52	17.1	75	12.0
**21**	Phac	Val-NH_2_	646.42/647.34	19.5	105	12.0
**22**	Phac	Tle-NH_2_	660.44/661.35	21.2	140	6.4
**23**	Phac	Pro-NH_2_	644.4/645.36	18.6	200	5.2
**24**	Phac	Sar-NH_2_	618.39/619.37	17.3	50	3.2
**25**	Phac	Gaba-NH_2_	632.41/633.29	17.1	15	26.3
**26**	Phac	Aca-NH_2_	660.44/661.37	18.9	17	n.d.
**27**	Phac	NH_2_	547.35/548.3	16.7	2.47	20.8
**28**	Phac	OH	548.34/549.4	17.3	27	n.d.
**29**	3,4-Cl_2_-Phac	NH_2_	615.28/616.13	24.8	1.13	25.1
**30**	3,4-Cl_2_-Phac	Gly-NH_2_	672.3/673.22	24.4	1.8	45.0
**31**	3-NH_2_-Phac	NH_2_	562.37/563.35	9.0	1.2	27.5
**32**	3,4-(Methylenedioxy)-Phac	NH_2_	591.35/592.31	17.4	0.68	19.9
**33**	4-OH-Phac	NH_2_	563.35/564.45	13.6	0.54	27.5
**34**	4-Phenyl-Phac	NH_2_	623.39/624.31	27.9	0.53	27.1
**35**	4-AMe-Phac	NH_2_	576.39/577.36	10.9	0.38	31.7
**36**	3-AMe-Phac	NH_2_	576.39/577.19	10.9	0.36	12.4
**37**	3-GMe-Phac	NH_2_	618.41/619.36	12.7	0.18	32.3
**38**	4-GMe-Phac	NH_2_	618.41/619.32	12.4	0.11	65.0
**39**	d/l-α-phenylglycine	NH_2_	562.37/563.30	10.0/10.8	3.6	n.d.
**40**	Ac-d/l-α-phenylglycine	NH_2_	604.38/605.31	14.5/14.9	4.18	n.d.
**41**	Cyclohexylacetic acid	NH_2_	553.41/554.6	20.0	4.8	36.7
**42**	α-Cyclohexylglycine	NH_2_	568.42/569.66	12.4	26.3	31.7
**43**	1-Adamantylacetic acid	NH_2_	605.44/606.37	16.9	13.8	24.2
**44**	1-Adamantylglycine	NH_2_	620.45/621.2	17.3	70.0	14.6

a See Figure 2 for abbreviation; n.d.: not determined.

b % Inhibition at 100 μM inhibitor in the presence of 125 μM substrate.

For further elongation of the peptide backbone, the P4 phenylacetyl residue was replaced by the structurally related phenylglycine containing an additional amino group. However, during HPLC analysis of product **39** and of its acetylated analog **40**, two peaks with similar area and retention time were obtained for each compound (Table 2), which also provided identical mass-to-charge ratios. This suggested a racemization of the Cα-atom of the phenylglycines, which is known from the literature and mainly occurs during standard Fmoc deprotection^23^. For both racemic inhibitors, reduced *K*_i_ values around 4 µM have been determined. To overcome the racemization problem, the structurally related non-aromatic α-cyclohexylglycine was used as P4 group. A relatively weak potency was observed for the cyclohexylglycine inhibitor **42**, as well as for the sterically more demanding 1-adamantylglycine compound **44**. For their des-amino analogs **41** and **43**, an approximately 5-fold improved potency against the WNV protease was determined, although these inhibitors are less potent than the phenylacetyl derivatives.

Most tested derivatives showed a relatively weak inhibition of the DENV enzyme. Similarly, for the WNV protease, the strongest potency (65% inhibition at 100 µM inhibitor concentration) was found for the P4 *p*-guanidinomethyl-phenylacetyl compound **38**.

Based on the strong potency of compound **38** for the WNV enzyme, we have also prepared its P1 GCMA analog 4-GMe-Phac-Lys-Lys-GCMA, which inhibits the WNV protease with a *K*_i_ value of 0.23 µM, but suffers from poor potency against the DENV protease (*K*_i_ = 67 µM).

### Chimeric furin and flavivirus protease inhibitors

Aside from the flavivirus proteases, host enzymes are also involved in the maturation of the viral proteins. For instance, the PC furin performs the essential cleavage of the viral prM protein^30^. The similar preference of the viral proteases and of furin for multibasic substrates suggested to test some of our previously described furin inhibitors against the WNV and DENV proteases as well. However, only moderate activities were found for our most potent 4-amidinobenzylamine-derived furin inhibitors **45** and **46**, available from previous studies (Table 3)^24^^,^^25^. Their affinities could be significantly improved by replacement of the aliphatic P3 residues with arginine and lysine in compounds **47** and **48**, respectively. The Lys inhibitor **48** is the only compound possessing low micromolar affinity against the DENV protease.

### Inhibition of virus propagation in cell culture

All tested inhibitors were found to be non-toxic toward Huh-7 cells, a negligible influence on cell viability was found up to concentrations of 50 µM using the CellTiter-Glo^®^ and the CytoTox 96^®^ assays, which measure the amount of ATP present and the release of lactate dehydrogenase, respectively (Promega GmbH, data not shown).

**Figure 4.**
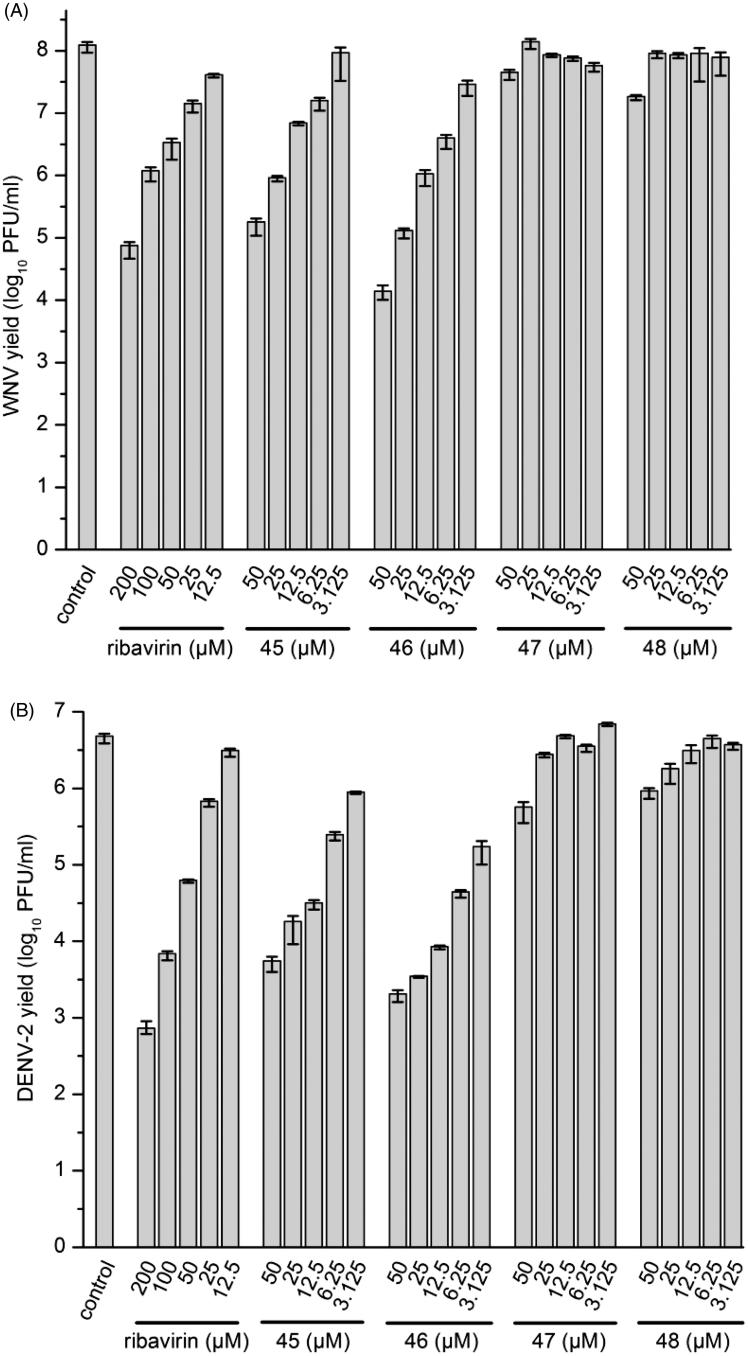
Reduction of WNV (A) and DENV-2 (B) propagation by inhibitors **45**–**48**. The number of formed infectious viruses (virus yield ± standard deviation, *n* = 3) was determined at 48 h postinfection from the supernatant of infected cells by a plaque assay. The nucleoside analog ribavirin was used as a reference.

Selected inhibitors were assessed for antiviral activity in a cell-based system using a plaque assay^20^^,^^28^. In this assay, production of infectious virus progeny depends on both the viral protease and host proteases like furin. We observed a pronounced concentration-dependent antiviral effect for some of the chimeric furin and flavivirus protease inhibitors, especially for compound **46**. At the highest inhibitor concentration of 50 µM, the number of infectious WNV particles was reduced by four orders of magnitude, while a less than 10-fold reduction (decrease by ∼75%) of virus titers was determined at the lowest concentration of 3.1 µM (Figure 4(a)). A similar tendency was observed against DENV-2. At 50 µM, a more than 1000-fold reduction was found and still a 10-fold decrease at 3.1 µM (Figure 4(b)). A slightly weaker effect was determined for analog **45**, which is also a very potent furin and moderate WNV and DENV protease inhibitor. At identical concentrations (e.g. 25 µM), both compounds provided a significantly stronger antiviral effect compared with the reference drug ribavirin. However, only a marginal antiviral effect was found for compounds **47** and **48** at the highest used concentrations, although they are still picomolar furin inhibitors with submicromolar efficacy against the flavivirus protease (Table 3). All of these results suggest that the antiviral effect of these compounds against WNV and DENV-2 is only based on furin inhibition.

## Discussion

All of the approved orally available inhibitors against thrombin, factor Xa, DPP IV, ACE, enkephalinase, the proteasome, renin and proteases of HIV and HCV belong to the group of active site directed inhibitors addressing relatively well defined binding pockets. In contrast, the WNV and DENV proteases, which reside on the cytosolic side of the endoplasmatic reticulum^9^, possess relatively flat active sites with a strong preference for polar multibasic substrates. Therefore, most substrate-analog inhibitors contain at least two or even three basic residues to achieve potency, though polar groups hamper membrane permeability and efficacy in cell culture assays. In preliminary studies with DENV-infected cells, no antiviral effect was found with our previously described protease inhibitors including compound **1** containing the GCMA residue in the P1 position. On the other hand, there exist various multibasic cell-penetrating peptides. It is assumed that they initially bind to negatively charged phospholipids on the cell surface, followed by membrane penetration or endocytotic uptake and subsequent escape from the endosomes^31^^,^^32^. This encouraged us to synthesize new compounds maintaining the tribasic inhibitor structure, because the replacement of Lys in the P3 position by non-basic amino acids was not accepted in a previous study and provided inhibitors with significantly reduced potency^18^. The relatively exposed and shallow binding site observed in the crystal structure of compound **1** in complex with the WNV protease, especially in the S3 region, argued that replacement of the flexible lysine residues by more rigid and bulky basic phenylalanine derivatives should increase potency. Similar replacements by incorporation of *para*- and *meta*-amidino- or guanidinophenylalanines have been recently described by the Klein group in a series of peptidomimetic inhibitors containing a C-terminal phenylglycine-amide in the P1 position^20^. At present, we cannot explain why all compounds summarized in Table 1 possessed reduced inhibitory potency against the WNV protease and negligible activity against the DENV enzyme. It might be possible that the binding of more bulky inhibitors is hindered by the ∼15 amino acid long peptide segment that contains the artificial (Gly)_4_-Ser-(Gly)_4_ linker part connecting NS2B with NS3, which cannot be seen in the published crystal structures. Especially, Pro91, the last visible residue of NS2B in the crystal structure of the WNV protease in complex with inhibitor **1**^19^, is located near the S3 pocket (distance to the P3 side-chain amino group ∼7.5 Å). It is conceivable that the following Gly-Ala-Pro-Trp-Ala segment of NS2B partially covers the active site thereby disturbing the binding of larger P3 and P2 residues.

Due to the reduced potency of these compounds, lysine was maintained as P2 and P3 residues in two additional inhibitor series. The replacement of GCMA by Arg in the P1 position enabled a convenient synthesis of these compounds by SPPS and allowed further modifications of the P4 residue, as well as a C-terminal elongation of the inhibitors. The effects of the primed site modifications confirm previous results described by the Strongin group, who had found a strong preference of the WNV protease for glycine in P1′ and P2′ positions^13^. The use of the other tested amino acids in the P1′ position was not accepted. Even the incorporation of Ala in inhibitor **19** resulted in a ∼10-fold drop in inhibitory potency against the WNV enzyme compared with the Gly analog **18**, although Ala16 is well accepted as P1′ residue in the complex with aprotinin^33^. Comparison of the crystal structures of the WNV protease revealed a relatively closed primed site in complex with inhibitor **1** due to the formation of a weak hydrogen bond between the NH of Ser135 and the carbonyl oxygen of Thr132 (distance 3.4 Å). In contrast, in the aprotinin complex (PDB: 2IJO^33^) the loop located at the N-terminal side of Ser135 adopts a slightly different conformation, in which Thr132 is moved away. Moreover, the carbonyl group of Thr132 points in the opposite direction, which prohibits the interaction with Ser135 and provides a more open primed site, which can accept Ala as P1′ residue in aprotinin.

A similar potency was observed with the arginine-amide inhibitor **27** lacking the primed-site glycines used in compounds **17** and **18**. Therefore, this more simple structure was used for further modifications of the P4 residue. The strongest affinity was found for the tetrabasic inhibitors **37** and **38**, which possess *K*_i_ values <0.2 µM for the WNV protease. However, the relatively small differences in affinity compared to the inhibitors with neutral P4 group (e.g. compounds **29**–**33**) suggest that the P4 *para*- or *meta*-guanidinomethyl substitution is not involved in specific polar contacts to the WNV protease. Assuming a similar binding mode as found for inhibitor **1**, the basic P4 group should be rather directed into the solvent. It might be possible that the additional basic P4 substituent only contributes to a faster electrostatically driven association resulting in a slightly improved inhibition constant. Such an effect was previously observed during kinetic analysis of the thrombin–hirudin interaction^34^. Single mutations of various glutamic acid residues in the C-terminal segment of hirudin, which binds to the multibasic anion-binding site-I of thrombin, predominantly reduced the association rate constant *k*_on_, thereby resulting in a weaker inhibition. In contrast, the dissociation rate constant *k*_off_ was less affected, because most of the acidic glutamic acid side-chains are not involved in direct interactions with thrombin as found by X-ray crystallography^35^.

The incorporation of phenylglycine, the α-amino-analog of the phenylacetyl group, enables a convenient elongation of the peptides. However, due to racemization problems of this residue in Fmoc-SPPS^23^ during the synthesis of inhibitors **39** and **40**, it was further replaced by the more hydrophobic cyclohexylglycine and the bulky 1-adamantylglycine. Since both inhibitors **42** and **44** suffered from weak potency when compared to their des-amino analogs **41** and **43** and to the phenylglycine inhibitors, no further elongated analogs have been prepared.

So far, most approved antiviral drugs predominantly target virus proteins without or only with weak effects on host enzymes and mechanisms, thereby ensuring a sufficient therapeutic index. However, such direct-acting antiviral drugs are limited by the development of drug resistance and the narrow spectrum covering most often just one virus species. To overcome these problems, a lot of effort has been invested to inhibit host targets involved in virus replication. This approach should overcome the problem of drug resistance, but depending on the specific host target could lead to more pronounced side effects. Based on our known furin inhibitors **45** and **46**, we prepared new analogs that should combine inhibition of the viral protease and the cellular enzyme furin. However, only a marginal efficacy was found for compounds **47** and **48** in WNV- and DENV-infected cell culture, although they are stronger inhibitors of the viral proteases *in vitro*. The significant activities of compounds **45** and **46**, which are more potent furin inhibitors, suggest that their antiviral properties solely depend on furin inhibition. So far, we could not identify any antiviral activity via inhibition of the WNV- and DENV-proteases in cell culture by the prepared multibasic active-site inhibitors. Most likely, this is caused by their negligible cell membrane permeability, which prevents the inhibition of proteases in the cytosol.

During the past few years, allosteric inhibitors of the WNV^36^^,^^37^ and DENV-proteases^38^ have been described. The development of such compounds might be a promising alternative especially for enzymes with poorly defined active sites. Besides the inhibition of the proteases, many other viral targets can be addressed; these strategies were recently reviewed^8^^,^^39^. An alternative approach could be the inhibition of host factors as shown here with our furin inhibitors, which reduced the WNV- and DENV replication in cell culture by several orders of magnitude.

## References

[1] Bhatt S, Gething PW, Brady OJ, et al The global distribution and burden of dengue. Nature 2013;496:504–7. 23563266 10.1038/nature12060 PMC3651993

[2] Suthar MS, Diamond MS, Gale M Jr. West Nile virus infection and immunity. Nat Rev Microbiol 2013;11:115–28. 23321534 10.1038/nrmicro2950

[3] Thomas SJ, Rothman AL. Trials and tribulations on the path to developing a dengue vaccine. Vaccine 2015;33:D24–31. 26122583 10.1016/j.vaccine.2015.05.095

[4] Hadinegoro SR, Arredondo-Garcia JL, Capeding MR, et al Efficacy and long-term safety of a dengue vaccine in regions of endemic disease. N Engl J Med 2015;373:1195–206. 26214039 10.1056/NEJMoa1506223

[5] Villar L, Dayan GH, Arredondo-Garcia JL, et al Efficacy of a tetravalent dengue vaccine in children in Latin America. N Engl J Med 2015;372:113–23. 25365753 10.1056/NEJMoa1411037

[6] Vannice KS, Durbin A, Hombach J. Status of vaccine research and development of vaccines for dengue. Vaccine 2016;34:2934–8. 26973072 10.1016/j.vaccine.2015.12.073

[7] Lim SP, Wang QY, Noble CG, et al Ten years of dengue drug discovery: progress and prospects. Antiviral Res 2013;100:500–19. 24076358 10.1016/j.antiviral.2013.09.013

[8] Lim SP, Shi PY. West Nile virus drug discovery. Viruses 2013;5:2977–3006. 24300672 10.3390/v5122977 PMC3967157

[9] Luo D, Vasudevan SG, Lescar J. The flavivirus NS2B-NS3 protease-helicase as a target for antiviral drug development. Antiviral Res 2015;118:148–58. 25842996 10.1016/j.antiviral.2015.03.014

[10] Nitsche C, Holloway S, Schirmeister T, Klein CD. Biochemistry and medicinal chemistry of the dengue virus protease. Chem Rev 2014;114:11348–81. 25268322 10.1021/cr500233q

[11] Steuber H, Kanitz M, Ehlert FR, Diederich W. Recent advances in targeting Dengue and West Nile virus proteases using small molecule inhibitors In: Diederich WE, Steuber H, eds. Therapy of viral infections. Topics in medicinal chemistry. Vol. 15 Berlin, Heidelberg: Springer; 2015:93–141.

[12] Chappell KJ, Stoermer MJ, Fairlie DP, Young PR. Insights to substrate binding and processing by West Nile Virus NS3 protease through combined modeling, protease mutagenesis, and kinetic studies. J Biol Chem 2006;281:38448–58. 17052977 10.1074/jbc.M607641200

[13] Shiryaev SA, Ratnikov BI, Aleshin AE, et al Switching the substrate specificity of the two-component NS2B-NS3 flavivirus proteinase by structure-based mutagenesis. J Virol 2007;81:4501–9. 17301157 10.1128/JVI.02719-06 PMC1900165

[14] Seidah NG, Prat A. The biology and therapeutic targeting of the proprotein convertases. Nat Rev Drug Discov 2012;11:367–83. 22679642 10.1038/nrd3699

[15] Lindenbach BD, Murray CL, Thiel H-J, Rice CM. Chapter 25: Flaviviridae In: David M, Knipe PMH, eds. Fields virology. 6th ed. Philadelphia, PA: Lippincott Williams & Wilkins; 2013:712–46.

[16] Shiryaev SA, Ratnikov BI, Chekanov AV, et al Cleavage targets and the d-arginine-based inhibitors of the West Nile virus NS3 processing proteinase. Biochem J 2006;393:503–11. 16229682 10.1042/BJ20051374 PMC1360700

[17] Lim HA, Joy J, Hill J, San Brian Chia C. Novel agmatine and agmatine-like peptidomimetic inhibitors of the West Nile virus NS2B/NS3 serine protease. Eur J Med Chem 2011;46:3130–4. 21565434 10.1016/j.ejmech.2011.04.055

[18] Lim HA, Ang MJ, Joy J, et al Novel agmatine dipeptide inhibitors against the West Nile virus NS2B/NS3 protease: a P3 and N-cap optimization study. Eur J Med Chem 2013;62:199–205. 23353753 10.1016/j.ejmech.2012.12.043

[19] Hammamy MZ, Haase C, Hammami M, et al Development and characterization of new peptidomimetic inhibitors of the West Nile virus NS2B-NS3 protease. ChemMedChem 2013;8:231–41. 23307694 10.1002/cmdc.201200497

[20] Weigel LF, Nitsche C, Graf D, et al Phenylalanine and phenylglycine analogues as arginine mimetics in dengue protease inhibitors. J Med Chem 2015;58:7719–33. 26367391 10.1021/acs.jmedchem.5b00612

[21] Saupe SM, Steinmetzer T. A new strategy for the development of highly potent and selective plasmin inhibitors. J Med Chem 2012;55:1171–80. 22276953 10.1021/jm2011996

[22] Bernatowicz MS, Wu Y, Matsueda GR. Urethane protected derivatives of 1-guanylpyrazole for the mild and efficient preparation of guanidines. Tetrahedron Lett 1993;34:3389–92.

[23] Elsawy MA, Hewage C, Walker B. Racemisation of N-Fmoc phenylglycine under mild microwave-SPPS and conventional stepwise SPPS conditions: attempts to develop strategies for overcoming this. J Pept Sci 2012;18:302–11. 22451378 10.1002/psc.2398

[24] Becker GL, Lu Y, Hardes K, et al Highly potent inhibitors of proprotein convertase furin as potential drugs for treatment of infectious diseases. J Biol Chem 2012;287:21992–2003. 22539349 10.1074/jbc.M111.332643 PMC3381159

[25] Hardes K, Becker GL, Lu Y, et al Novel furin inhibitors with potent anti-infectious activity. ChemMedChem 2015;10:1218–31. 25974265 10.1002/cmdc.201500103

[26] D’Arcy A, Chaillet M, Schiering N, et al Purification and crystallization of dengue and West Nile virus NS2B-NS3 complexes. Acta Crystallogr F Struct Biol Cryst Commun 2006;62:157–62. 10.1107/S1744309106001199 PMC2150946 16511290

[27] Dixon M. The determination of enzyme inhibitor constants. Biochem J 1953;55:170–1. 13093635 10.1042/bj0550170 PMC1269152

[28] Nitsche C, Schreier VN, Behnam MA, et al Thiazolidinone-peptide hybrids as Dengue Virus protease inhibitors with antiviral activity in cell culture. J Med Chem 2013;56:8389–403. 24083834 10.1021/jm400828u

[29] Stoermer MJ, Chappell KJ, Liebscher S, et al Potent cationic inhibitors of West Nile virus NS2B/NS3 protease with serum stability, cell permeability and antiviral activity. J Med Chem 2008;51:5714–21. 18729351 10.1021/jm800503y

[30] Heinz FX, Stiasny K. Flaviviruses and their antigenic structure. J Clin Virol 2012;55:289–95. 22999801 10.1016/j.jcv.2012.08.024

[31] Fuchs SM, Raines RT. Internalization of cationic peptides: the road less (or more?) traveled. Cell Mol Life Sci 2006;63:1819–22. 16909213 10.1007/s00018-006-6170-z PMC2812862

[32] Milletti F. Cell-penetrating peptides: classes, origin, and current landscape. Drug Discov Today 2012;17:850–60. 22465171 10.1016/j.drudis.2012.03.002

[33] Aleshin AE, Shiryaev SA, Strongin AY, Liddington RC. Structural evidence for regulation and specificity of flaviviral proteases and evolution of the Flaviviridae fold. Protein Sci 2007;16:795–806. 17400917 10.1110/ps.072753207 PMC2206648

[34] Braun PJ, Dennis S, Hofsteenge J, Stone SR. Use of site-directed mutagenesis to investigate the basis for the specificity of hirudin. Biochemistry 1988;27:6517–22. 3146347 10.1021/bi00417a048

[35] Rydel TJ, Tulinsky A, Bode W, Huber R. Refined structure of the hirudin–thrombin complex. J Mol Biol 1991;221:583–601. 1920434 10.1016/0022-2836(91)80074-5

[36] Samanta S, Lim TL, Lam Y. Synthesis *in vitro* evaluation of West Nile virus protease inhibitors based on the 2-{6-[2-(5-phenyl-4H-{1,2,4]triazol-3-ylsulfanyl)acetylamino]benzothiazol-2-ylsul fanyl}acetamide scaffold. ChemMedChem 2013;8:994–1001. 23619931 10.1002/cmdc.201300114

[37] Gao Y, Samanta S, Cui T, Lam Y. Synthesis *in**vitro* evaluation of West Nile virus protease inhibitors based on the 1,3,4,5-tetrasubstituted 1H-pyrrol-2(5H)-one scaffold. Chem Med Chem 2013;8:1554–60. 23868614 10.1002/cmdc.201300244

[38] Wu H, Bock S, Snitko M, et al Novel Dengue Virus NS2B/NS3 protease inhibitors. Antimicrob Agents Chemother 2015;59:1100–9. 25487800 10.1128/AAC.03543-14 PMC4335830

[39] Behnam MA, Nitsche C, Boldescu V, Klein CD. The medicinal chemistry of dengue virus. J Med Chem 2016;59:5622–49. 26771861 10.1021/acs.jmedchem.5b01653

